# β-Glucan-stimulated neutrophil secretion of IL-1α is independent of GSDMD and mediated through extracellular vesicles

**DOI:** 10.1016/j.celrep.2021.109139

**Published:** 2021-05-18

**Authors:** Bridget Ratitong, Michaela Marshall, Eric Pearlman

**Affiliations:** 1 Department of Physiology and Biophysics, University of California, Irvine, Irvine, CA, USA; 2 Department of Ophthalmology, University of California, Irvine, Irvine, CA, USA; 3 Institute for Immunology, University of California, Irvine, Irvine, CA, USA; 4 Lead contact

## Abstract

Neutrophils are an important source of interleukin (IL)-1β and other cytokines because they are recruited to sites of infection and inflammation in high numbers. Although secretion of processed, bioactive IL-1β by neutrophils is dependent on NLRP3 and Gasdermin D (GSDMD), IL-1α secretion by neutrophils has not been reported. In this study, we demonstrate that neutrophils produce IL-1α following injection of *Aspergillus fumigatus* spores that express cell-surface β-Glucan. Although IL-1α secretion by lipopolysaccharide (LPS)/ATP-activated macrophages and dendritic cells is GSDMD dependent, IL-1α secretion by β-Glucan-stimulated neutrophils occurs independently of GSDMD. Instead, we found that bioactive IL-1α is in exosomes that were isolated from cell-free media of β-Glucan-stimulated neutrophils. Further, the exosome inhibitor GW4869 significantly reduces IL-1α in extracellular vesicles (EVs) and total cell-free supernatant. Together, these findings identify neutrophils as a source of IL-1α and demonstrate a role for EVs, specifically exosomes, in neutrophil secretion of bioactive IL-1α.

## In brief

Neutrophils have functional NLRP3 and NLRC4 and are recognized as an important source of IL-1β. Ratitong et al. demonstrate that murine neutrophils also produce IL-1α. Unlike macrophages, neutrophil IL-1α is secreted in extracellular vesicles and is released independently of gasdermin D and cell death.

## Graphical Abstract

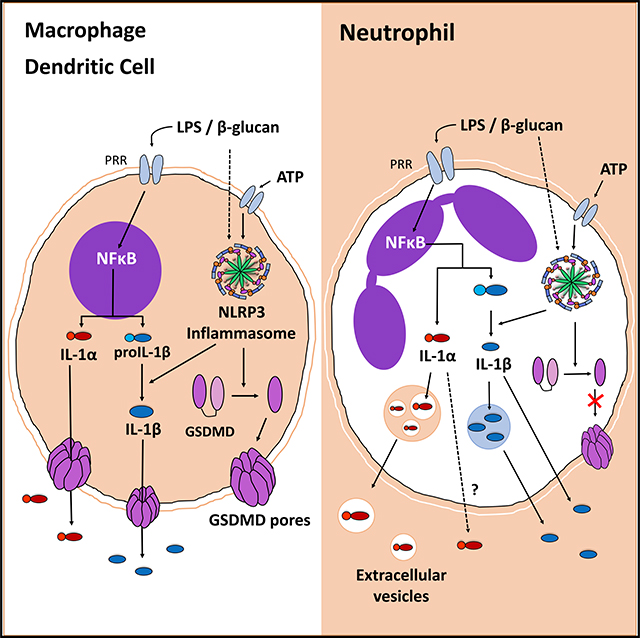

## INTRODUCTION

Interleukin (IL)-1α and IL-1β are pro-inflammatory cytokines that lack the signal peptide for endoplasmic reticulum (ER)/Golgi-dependent secretion and are released by non-canonical pathways (Rubartelli et al., 1990). Canonical IL-1β secretion by murine macrophages is tightly regulated in a two-step process: signal 1 is induced through pathogen recognition receptors leading to transcription of pro-IL-1α and pro-IL-1β. For IL-1β, a second signal, such as ATP activation of the P2X7 receptor, is required for assembly of the multi-protein NLRP3 inflammasome complex, which mediates caspase-1 processing of pro-IL-1β to its bioactive form. Caspase-1 also cleaves pro-Gasdermin D (GSDMD) to NGSDMD subunits that rapidly assemble and form pores in the plasma membrane, leading to passive IL-1β release and pyroptotic cell death (Broz et al., 2020; Evavold et al., 2018; Shi et al., 2015). Although secretion through GSDMD pores appears to be the most rapid means of IL-1β release, GSDMD-independent mechanisms of IL-1β secretion have been reported, including trafficking to the plasma membrane and release through PIP_2_-rich membrane microdomains (Monteleone et al., 2018) and secretory autophagy in which autophagosomes containing mature IL-1β are trafficked to the plasma membrane instead of lysosomes (Claude-Taupin et al., 2018; Kimura et al., 2017).

Neutrophils are also an important source of IL-1β because they are recruited in large numbers to sites of infection and inflammation. Although neutrophils have functional NLRP3 and NLRC4 inflammasomes, unlike macrophages, inflammasome activation in neutrophils does not result in pyroptosis (Chen et al., 2014; Karmakar et al., 2016; Kovacs et al., 2020). Further, whereas GSDMD is required for IL-1β secretion by neutrophils, N-GSDMD does not localize to the plasma membrane; instead, it is detected in the membrane of primary granules and autophagosomes, and autophagy-related proteins are required for IL-1β secretion (Karmakar et al., 2020).

In contrast to IL-1β, there are relatively few studies on IL-1α in infection or inflammation. In contrast to IL-1β, IL-1α is bioactive in both the pro-form and following cleavage by calpain (Di Paolo, 2016; Malik, 2018). Gross et al. (2012) reported that in a murine model of monosodium urate (MSU)-induced peritonitis, neutrophil recruitment to the peritoneal cavity was significantly impaired in *Il1a*^−/−^ mice. Similarly, Caffrey et al. (2015) demonstrated impaired neutrophil recruitment to the lungs of *Il1a*^−/−^ mice in a model of pulmonary aspergillosis (Caffrey-Carr et al., 2017).

In the current study, we found an important role for IL-1α in neutrophil recruitment using a fungal peritonitis model where *A. fumigatus* conidia (spores) are injected into the peritoneal cavity. Further, we show that neutrophils are a source of IL-1α during inflammation. We found that whereas IL-1α secretion by bone marrow (BM)-derived dendritic cells (BMDCs) and macrophages is dependent on GSDMD, IL-1α secretion by β-Glucan-stimulated neutrophils is mediated through extracellular vesicles (EVs), primarily exosomes. Together, these findings identify neutrophils as a source of IL-1α and define a non-canonical pathway for IL-1α secretion.

## RESULTS

### Neutrophil recruitment is dependent on IL-1α, and neutrophils are a source of IL-1α in *A. fumigatus*-induced peritonitis

**Figure 1.**
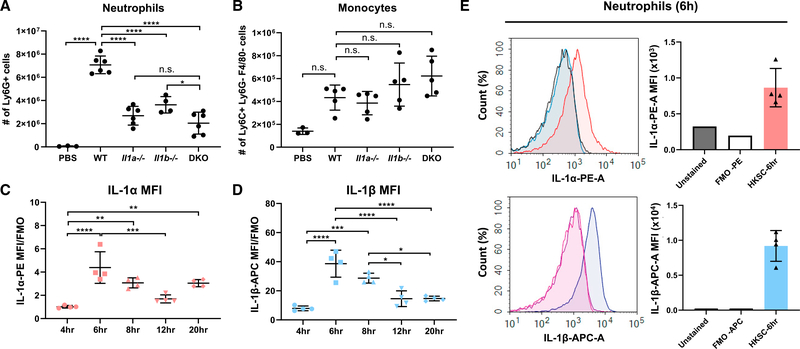
Neutrophils are a source of IL-1α in *A. fumigatus*-induced peritonitis (A and B) Neutrophils (A) and monocytes (B) were quantified by flow cytometry 24 h after i.p. injection of heat-killed *A. fumigatus* conidia into WT, IL-1α^−/−^, IL-1β^−/−^, or IL-1α^−/−^/IL-1β^−/−^ DKO mice. Cell numbers shown are percentage of each cell type 3 total cell count. (C and D) MFI of intracellular IL-1α (C) and IL-1β (D) in neutrophils from the peritoneal cavity at multiple time points after injection of A. fumigatus conidia. IL-1α MFI and IL-1β MFI were normalized to FMO control (n = 4). (E) Representative histogram and corresponding MFI levels after 6-h incubation (n = 4). Each data point represents an individual mouse. Experiments were repeated twice with similar results. Significance was calculated by one-way ANOVA followed by Dunnett’s multiple comparisons test. *p < 0.05, **p < 0.01, ***p < 0.001, ****p < 0.0001.

To examine the role of IL-1α in neutrophil recruitment, we repeated the experiments by Gross et al. (2012) and also found impaired neutrophil recruitment in *Il1a*^−/−^ and *Il1a/b*^−/−^ mice compared with C57BL/6 wild-type (WT) mice following intraperitoneal (i.p.) injection of MSU (2 mg/mouse); there was no difference in monocyte numbers (Figures S1A and S1B). Because we are interested in the response against fungal infection, we repeated the experiment using a model of peritonitis where 1 × 10^7^ *Aspergillus fumigatus* germinating conidia (spores) expressing cell-surface β-Glucan were heat killed and injected into the peritoneal cavity of WT, *Il1a*^−/−^, *Il1b*^−/−^, and *Il1a*^−/−^/ *Il1b*^−/−^ double-knockout (DKO) mice. Neutrophils, monocytes, and macrophages were quantified by flow cytometry after 4 h (gating strategy is shown in Figure S1C). We found that *A. fumigatus* conidia induced infiltration of 7 × 10^6^ neutrophils compared with 4 × 10^5^ monocytes, and that *Il1a*^−/−^, *Il1b*^−/−^, and DKO mice had significantly fewer neutrophils than WT mice (Figures 1A and 1B). In contrast to neutrophils, there were no significant differences in the number of monocytes and macrophages recruited to the peritoneal cavity of *Il1a*^−/−^, *Il1b*^−/−^, or DKO mice compared with WT (Figures 1B and S1D).

We next examined if neutrophils are a source of IL-1α by examining intracellular IL-1α and IL-1β following i.p. injection of *A. fumigatus* conidia. IL-1α and IL-1β mean fluorescence intensity (MFI) levels peaked at 6 h after injection (Figures 1C and 1D), and secreted IL-1α in the peritoneal lavage was highest at earlier time points (Figure S1E). Representative MFI of intracellular IL-1α and IL-1β in neutrophils at 6 h showed increased production of both cytokines over fluorescent minus one (FMO) control (Figure 1E).

Collectively, these data identify neutrophils as a source of IL-1α. Although IL-1β production was 10-fold higher than IL-1α, elimination of IL-1α yields a similar phenotype as IL-1β, indicating that even the relatively low levels of IL-1α are important in this model of inflammation.

### Distinct roles for GSDMD in IL-1α secretion by dendritic cells, macrophages, and neutrophils

**Figure 2.**
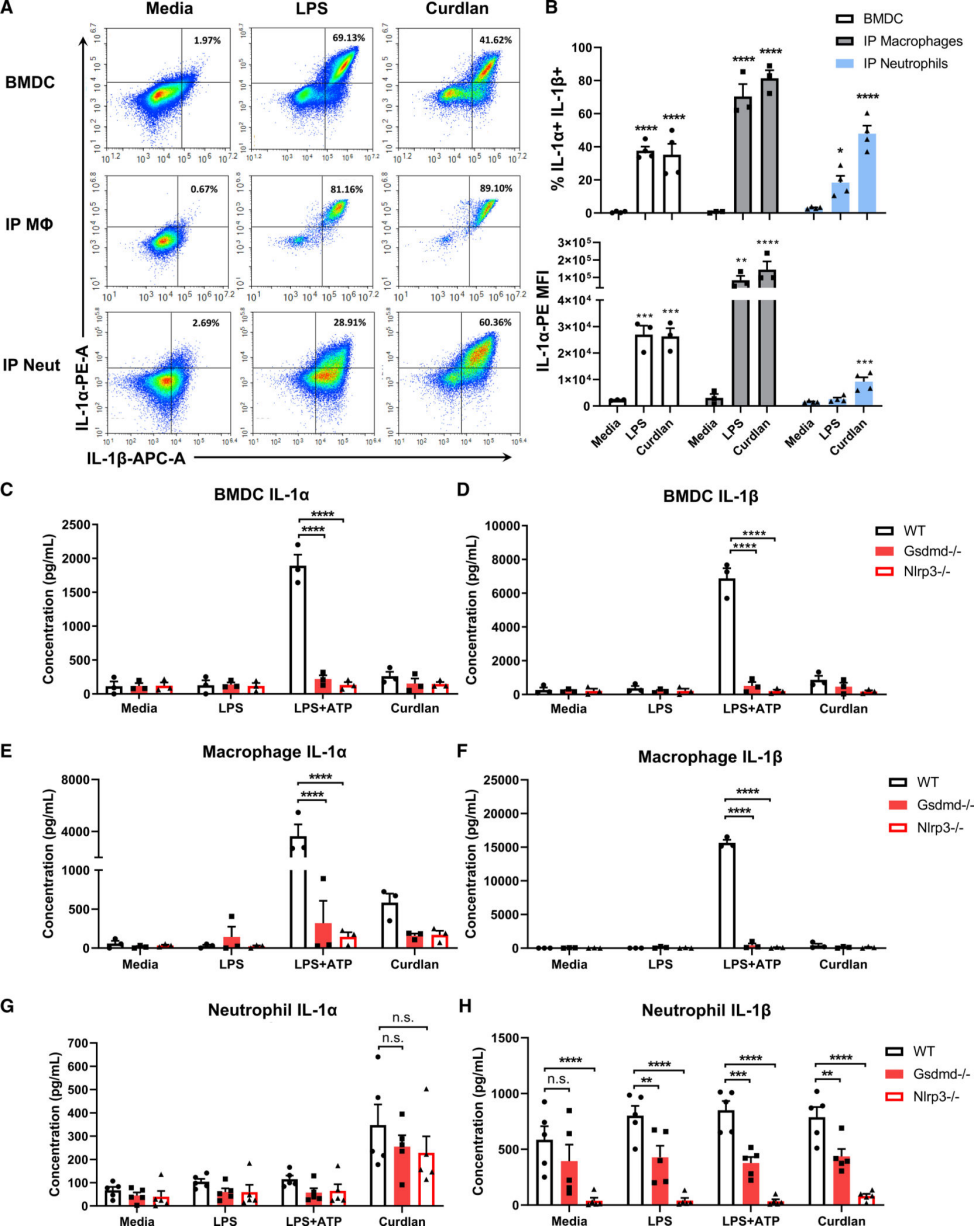
IL-1α secretion by bone-marrow-derived dendritic cells, peritoneal macrophages, and neutrophils (A) Representative flow cytometry plots of intracellular IL-1α and IL-1β in BMDCs, macrophages, and neutrophils following 6-h stimulation with LPS or β-Glucan (curdlan). (B) Quantification of percent IL-1α^+^/IL-1β^+^ cells (B) and IL-1α MFI (C) (n = mean of total cells in 3 independent experiments). (C–H) IL-1α and IL-1β secretion by BMDCs, macrophages, and neutrophils from WT, *Gsdmd*^−/−^, and *Nlrp3*^−/−^ mice. (C and D) FACS-isolated CD11c^+^ Ly6G^−^ F4/80^−^ BMDCs (n = 3), (E and F) peritoneal macrophages (≥95% F4/80^+^, n = 3), and (G and H) neutrophils (≥98% Ly6G^+^, n = 5). Significance was calculated by two-way ANOVA with Tukey’s multiple comparisons test. *p < 0.05, **p < 0.01, ***p < 0.001, ****p < 0.0001.

We next examined IL-1α production by neutrophils compared with macrophages and BMDCs. Cells were incubated for 6 h with lipopolysaccharide (LPS) or insoluble β-Glucan (curdlan). LPS and curdlan induced IL-1α^+^IL-1β^+^ populations of macrophages, BMDCs, and neutrophils, although curdlan was more effective at activating neutrophils (Figures 2A and 2B). MFI levels in neutrophils were lower than macrophages and BMDCs, indicating that neutrophils produce less IL-1α on a per-cell basis (Figure 2C). However, neutrophils comprise the majority of infiltrating cells in inflamed tissues and are therefore an important source of IL-1α under these conditions.

Because IL-1α is co-expressed with IL-1β in BMDCs, macrophages, and neutrophils (Figures 2A and 2B), we examined whether IL-1α secretion follows the same mechanism as IL-1β. To examine the role of GSDMD in IL-1α secretion, we quantified IL-1α secreted by WT, *Gsdmd*^−/−^, and *Nlrp3*^−/−^ cells following stimulation with LPS, LPS/ATP, or curdlan. Fluorescence-activated cell sorting (FACS)-isolated BMDCs (≥99%, CD11c^+^ F4/80^−^ Ly6G^−^) and i.p. macrophages (≥95% F4/80^+^) that were primed with LPS and stimulated with ATP induced high levels of IL-1α and IL-1β secretion in WT, but not *Gsdmd*^−/−^ or *Nlrp3*^−/−^, cells (Figures 2C–2F). BMDCs and macrophages secreted relatively low levels of IL-1α and IL-1β in response to curdlan compared with LPS+ATP stimulation.

In contrast to BMDCs and macrophages, enriched i.p. neutrophils (≥97%, Ly6G^+^) secreted higher levels of IL-1α in response to curdlan compared with LPS. Further, although IL-1β secretion by neutrophils was dependent on GSDMD and NLRP3, there was no significant difference in IL-1α secretion between WT, *Gsdmd*^−/−^, and *Nlrp3*^−/−^ cells (Figures 2G and 2H). Surprisingly, we found IL-1β production in the absence of additional stimulation (Figure 2I), which is likely a consequence of NLRP3 activation of neutrophils isolated from the peritoneal cavity following casein injection.

In contrast to IP neutrophils, <15% BM neutrophils had intracellular IL-1α^+^IL-1β^+^ and secreted <80 pg/mL IL-1α in response to LPS+ATP (Figures S2A and S2B). IL-1α secretion by BM neutrophils was GSDMD dependent; however, they were not responsive to curdlan. Consistent with reports that neutrophils recognize β-Glucan via the lectin binding domain of CR3 (CD18/CD11b) (Leal et al., 2010; O’Brien and Reichner, 2016), we found that BM neutrophils do not express plasma membrane CD18 and have lower levels of CD11b than i.p. neutrophils (Figures S2C and S2D). Consequently, BM neutrophils did not secrete IL-1α when stimulated with depleted zymosan or *A. fumigatus* hyphal extracts (Figure S2E).

Together, these data show that IL-1α secretion by LPS/ATP-stimulated BMDCs and macrophages is dependent on NLRP3 and GSDMD In contrast, β-Glucan-induced IL-1α secretion by CR3-expressing neutrophils is GSDMD independent.

### β-Glucan induces pro-GSDMD cleavage and increased membrane permeability, but not cell death

IL-1α is passively released through cell death in non-hematopoietic cells (England et al., 2014; Scarpa et al., 2015), and GSDMD-dependent IL-1β secretion by macrophages results in pyroptotic cell death (Shi et al., 2017). Therefore, we next examined GSDMD cleavage, membrane permeability, and pyroptotic cell death.

**Figure 3.**
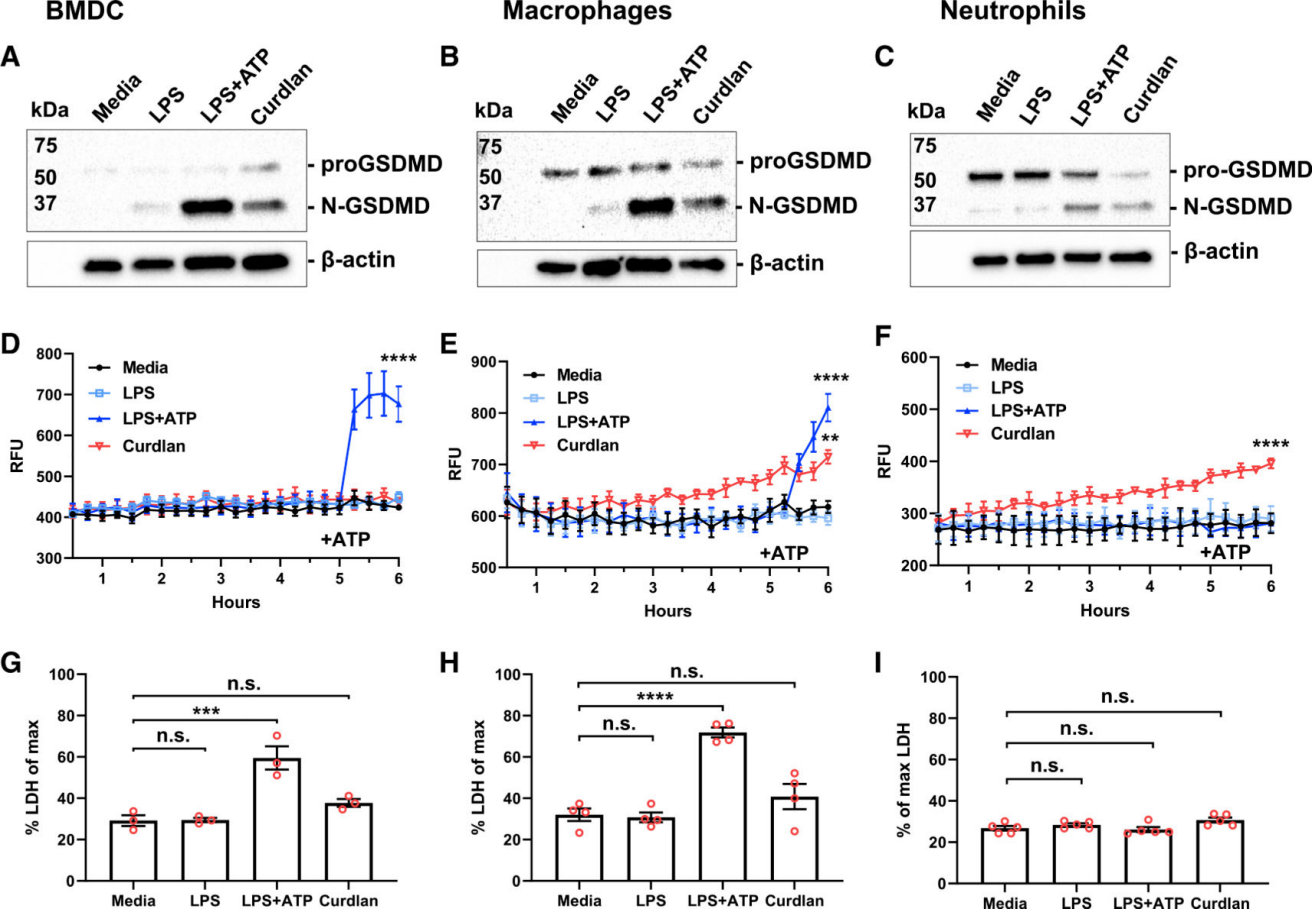
β-Glucan induced GSDMD cleavage and increased membrane permeability, but not cell death (A–C) Western blot analysis of GSDMD cleavage following 6-h stimulation with LPS, LPS/ATP, or curdlan in BMDCs (A), peritoneal macrophages (B), and peritoneal neutrophils (C). (D–F) Propidium iodide (PI) uptake measured over 6 h in each cell type. (G–I) Lactate dehydrogenase (LDH) release after 6-h incubation as a measure of cell death was calculated as percent of maximum (lysed cells). Two-way ANOVA with Tukey’s multiple comparisons test was used for PI uptake, and one-way ANOVA with Dunnett’s multiple comparisons test for LDH release. *p < 0.05, **p < 0.01, ***p < 0.001, ****p < 0.0001. Western blots are representative of three repeat experiments; PI and LDH data points represent 3–5 biological replicates.

BMDCs, i.p. macrophages, and i.p. neutrophils were examined by western blot for pro- and N-GSDMD. We found that in all cell types incubated with LPS+ATP or curdlan, pro-GSDMD was cleaved to the 31-kDa N-GSDMD (Figures 3A–3C). Consistent with GSDMD cleavage, propidium iodide (PI) uptake, indicative of plasma membrane permeability, was observed following ATP activation of LPS-primed BMDCs and macrophages, but not neutrophils (Figures 3D–3F). Although curdlan did not induce PI uptake in BMDCs, we observed a gradual increase in PI uptake in macrophages and neutrophils. Lactate dehydrogenase (LDH) release (indicative of cell lysis) was elevated in LPS/ATP, but not β-Glucan-stimulated DCs or macrophages (Figures 3G and 3H). However, there was no significant increase in LDH release by neutrophils under any of these conditions (Figure 3I). Consistent with previous reports, there was also no LDH release above background in *Gsdmd*^−/−^ or *Nlrp3*^−/−^ BMDCs, macrophages, or neutrophils (data not shown).

Collectively, these findings demonstrate that β-Glucan induces GSDMD cleavage in each of these cell types and increased plasma membrane permeability in macrophages and neutrophils. Despite GSDMD cleavage, there was no significant LDH release, indicating that β-Glucan-mediated IL-1α and IL-1β secretion occurs in the absence of pyroptotic cell death.

### Neutrophil IL-1α is released through EVs

Given that IL-1α release by stimulated neutrophils is independent of GSDMD and cell death, we examined other unconventional secretion pathways. EVs have emerged as an important mechanism for intercellular communication. Although most cells secrete EVs at steady state, their cargo and number depend on the stimulus (van Niel et al., 2018; Pegtel and Gould, 2019; Raposo and Stahl, 2019). EV-mediated cytokine release has been described in multiple cells and tissues (Fitzgerald et al., 2018). However, the role of EVs in mediating IL-1α secretion has not been clearly defined.

**Figure 4.**
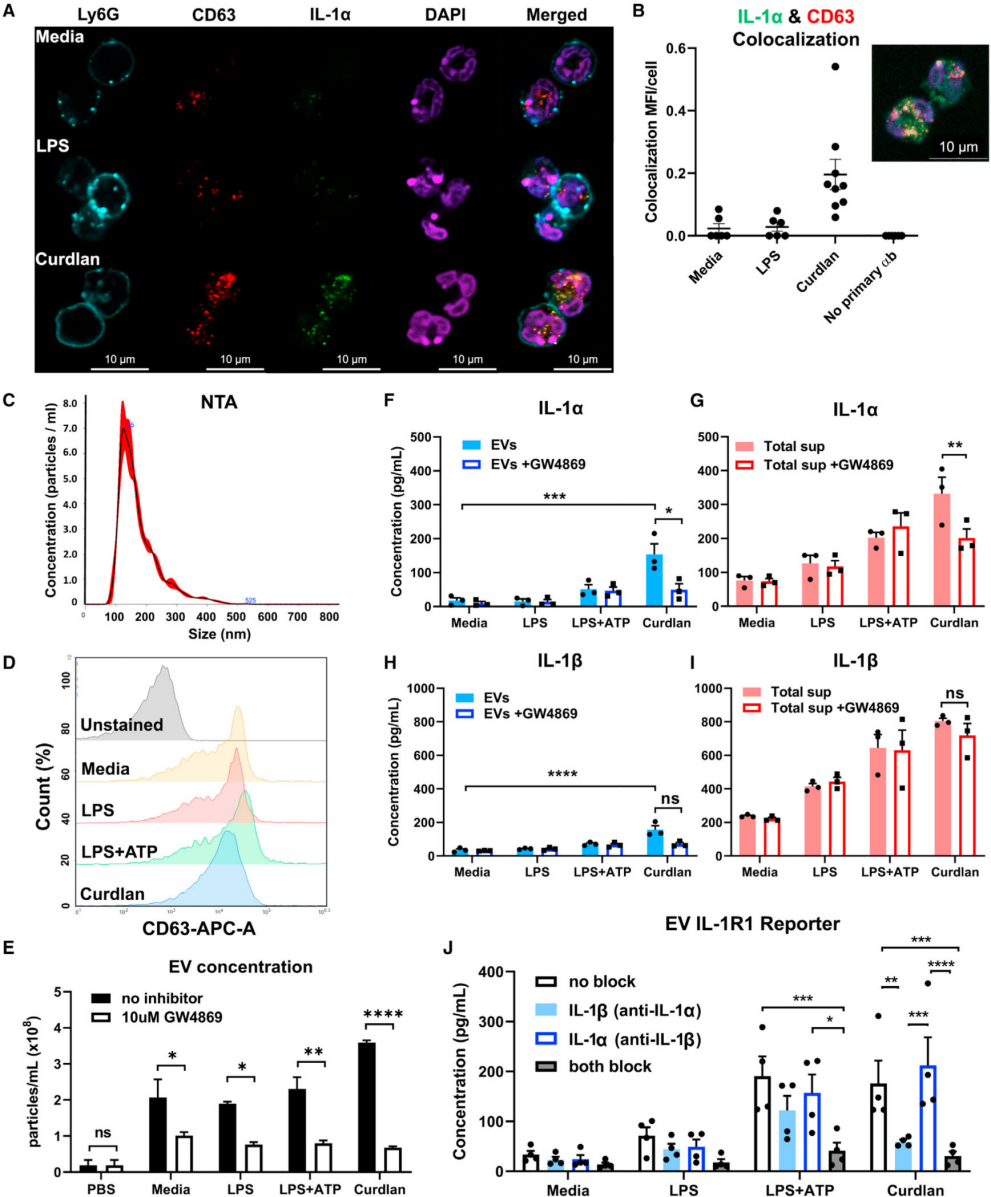
Exosomal release of IL-1α by neutrophils (A) Representative confocal images of peritoneal neutrophils stimulated with LPS or curdlan for 6 h. (B) Quantification of IL-1α and CD63 co-localization using ImageJ (each data point represents a single cell). (C) NTA of EV size distribution and concentration. (D–G) Neutrophils were stimulated in the presence of exosome inhibitor GW4869, and IL-1α and IL-1β were quantified by ELISA in isolated EVs following lysis (D and F) and in total cell-free supernatants (E and G). (H) Inhibition of EV secretion shown by NTA. (I) Bioactive IL-1 signaling through IL-1R1 reporter cells was measured in isolated exosomes in the absence of detergent lysis (n = 4). Neutralizing antibodies (Abs) to IL-1α, IL-1β, or both cytokines were included in the reporter assay, and bioactive cytokine concentration was calculated based on a standard curve using recombinant IL-1α and IL-1β. Two-way ANOVA with Tukey’s multiple comparisons test. *p < 0.05, **p < 0.01, ***p < 0.001, ****p < 0.0001. Experiments in (A) and (B) were repeated three times; (C)–(G) are biological replicates from repeat experiments.

To determine whether IL-1α is secreted in EVs, and specifically in exosomes, we first examined if IL-1α localizes with the exosome marker CD63 in stimulated neutrophils. Representative confocal microscopy images and quantification using ImageJ showed colocalization of IL-1α and CD63, most notably in curdlan-stimulated neutrophils (Figures 4A, 4B, and S3A). Imaging flow cytometry also revealed co-localization of IL-1α with CD63 in 17%–24% of stimulated neutrophils (Figure S3B).

Second, we examined isolated EVs using the ExoQuick-TC kit, which enriches for exosomes by co-precipitation with polymers. EVs were characterized by nanoparticle tracking analysis (NTA); surface expression of CD63, CD9, and CD81; and inhibition with GW4869. NTA has been used extensively to characterize and quantify EVs (Jung et al., 2020; Koritzinsky et al., 2017; Shao et al., 2018). NTA showed that most neutrophil-isolated EVs were within the size range of exosomes and small microvesicles (100–200 nm; Figure 4C). Flow cytometry also showed that exosome markers CD63, CD9, and CD81 were each expressed on isolated EVs from unstimulated and stimulated neutrophils, indicating that exosomes are a major component of this EV population (Figures 4D and S3C). However, IL-1α was not detected on the surface of EVs (Figure S3D). As a third approach, neutrophils were incubated with GW4869, a neutral sphingomyelinase inhibitor that effectively inhibits exosome release (Essandoh et al., 2015; Jiang et al., 2019; Sitrin et al., 2011) (Figure 4E). These findings indicate that exosomes are a major component of EVs secreted by neutrophils.

To determine whether EVs contain IL-1α and IL-1β, we lysed neutrophil EVs from LPS, LPS/ATP, or curdlan-stimulated neutrophils and quantified IL-1α and IL-1β by ELISA. We found both cytokines in lysed EVs and in total cell-free supernatant (containing intact EVs; Figures 4F–4I). EVs from curdlan-stimulated neutrophils had significantly higher levels of IL-1α and IL-1β compared with unstimulated neutrophils (Figures 4F and 4H). We also isolated EVs by ultracentrifugation (100,000 × *g*) and detected IL-1α and IL-1β in exosomes from curdlan-stimulated neutrophils (Figure S4A).

Pre-incubation with GW4869 resulted in significantly reduced IL-1α, but not IL-1β, secretion by curdlan-stimulated neutrophils, indicating that IL-1α is secreted in exosomes (Figures 4F–4I). IL-1α secretion in total supernatants and EVs of curdlan-stimulated neutrophils increased over 24 h, although there was also a small increase in neutrophil cell death at later time points (Figures S4B and S4C). We also found that neutrophils incubated with GW4869 exhibited no difference in intracellular IL-1α and IL-1β, indicating that this inhibitor selectively blocks IL-1α secretion, but not production (Figures S4D–S4F). Although we expected to find an increase in IL-1α, it is likely that the increase on a per-cell basis is minor and not reflected well by MFI.

Given that pro-IL-1α is bioactive, whereas IL-1β bioactivity requires processing, we next determined whether IL-1α and IL-1β in intact EVs are bioactive. Isolated EVs from stimulated neutrophils were incubated with HEK293T IL-1R1 reporter cells in the presence of neutralizing antibodies to IL-1α, IL-1β, or both. The concentration of bioactive IL-1 was calculated based on a standard curve using recombinant cytokines. We found that EV-encapsulated IL-1 can signal its surface receptor (Figure 4J). IL-1R1 activation by LPS/ATP-stimulated neutrophils was mediated by IL-1α and IL-1β, but curdlan-stimulated neutrophil EVs were primarily mediated by IL-1α. This finding implies that the IL-1β detected in curdlan-stimulated neutrophil EVs by ELISA was not bioactive.

Finally, we found that the cytokines CXCL1 and TNF-α, which are released through the canonical Golgi/ER secretion pathway, were not detected in isolated EVs, and that GW4869 had no inhibitory effect on their secretion (Figures S4G and S4H). Collectively, these findings identify a selective role for exosomes in secreting bioactive IL-1α.

## DISCUSSION

We and others reported that neutrophils are an important source of IL-1β during bacterial and fungal infections. In the current study, we found that neutrophils also produce IL-1α, and that IL-1α plays an important role in neutrophil recruitment to the peritoneal cavity following injection of *A. fumigatus* conidia expressing surface β-Glucan. Initial recruitment of neutrophils following i.p. injection of casein is likely due to pro-inflammatory and chemotactic cytokines produced by resident macrophages and epithelial cells. However, neutrophils are also a major source of IL-1α, and likely mediate further neutrophil infiltration in a feed-forward mechanism.

Consistent with the response to *A. fumigatus*, we found that particulate β-Glucan (curdlan) induced IL-1α secretion by i.p. neutrophils, whereas macrophages and BMDCs (and BM neutrophils) produced more IL-1α in response to LPS/ATP. We and others reported that neutrophils recognize β-Glucan by CR3 (CD11b/CD18) rather than Dectin-1 (Clark et al., 2018; Leal et al., 2010; O’Brien and Reichner, 2016). Here, we identified a key difference between BM and i.p. neutrophils where BM neutrophils lack a functional CR3 and do not respond to fungal products.

IL-1α secretion has mostly been studied as an alarmin that is released from non-hematopoietic cells following cell death (England et al., 2014). In contrast, there are relatively few reports on IL-1α secretion by myeloid cells. A recent study showed that *Staphylococcus aureus* can induce GSDMD-dependent IL-1α and IL-1β secretion by macrophages (Evavold et al., 2018). Here, we also found that IL-1α secretion by LPS/ATP-stimulated macrophages (and BMDCs) requires GSDMD. In contrast, IL-1α secretion by β-Glucan-stimulated i.p. neutrophils was independent of GSDMD. We reported that β-Glucan induces caspase-1- and caspase-11-dependent IL-1β secretion in the absence of cell death (Sun et al., 2018). Similarly, we found in the current study that β-Glucan did not induce cell death in any of these cell types, despite finding increased membrane permeability in macrophages and neutrophils.

Further, we show that instead of secretion through GSDMD pores, IL-1α secretion by β-Glucan-stimulated neutrophils is mediated by exosomes. Although co-localization of IL-1α with the tetraspanin CD63 measured by Amnis ImageStream was in only approximately 20% of neutrophils, this measures only complete localization and might have excluded cells with partial localization. Neutrophil heterogeneity may also contribute to the differences in cytokine production. Taken together with the presence of IL-1 in isolated exosomes, and that GW4869 inhibited IL-1α, but not IL-1β secretion, we conclude that exosomes are an important mechanism of IL-1α secretion by neutrophils. We also demonstrated using IL-1R1 reporter cells that exosomal IL-1α is bioactive.

Although IL-1β secretion was not inhibited by GW4869, it is possible that IL-1β is present in microvesicles given that isolation of exosomes can include other vesicles in the same size range. Consistent with this possibility, ATP activation of the P2X7 receptor on THP-1 monocytes induced secretion of microvesicles containing IL-1β (MacKenzie et al., 2001). Further, because cleaved IL-1β in macrophages triggers its relocation to PIP_2_-enriched plasma membrane domains (Monteleone et al., 2018), it is possible that membrane-associated IL-1β is released in microvesicles.

Because IL-1α was not detected on the surface of EVs, it is not clear how IL-1α activates IL-1R1. It is possible that encapsulated cytokines are released when EVs come close to their target cell as liposomes become leaky (Yang et al., 2010). Alternatively, human neutrophils secrete phospholipase A_2_ in response to fMLP stimulation (Degousee et al., 2002), which could degrade the EVs. Future studies will examine the role of phospholipases in this process.

In conclusion, our data clearly identify EVs as an important mechanism of IL-1α secretion by neutrophils. However, EVs are likely not the only mechanism by which this cytokine is secreted, and we will continue to examine additional pathways. Nonetheless, results from the current study clearly identified neutrophils as a source of IL-1α following injection of *A. fumigatus* conidia and add to our understanding of IL-1α regulation during the inflammatory process.

## STAR★METHODS

### RESOURCE AVAILABILITY

#### Lead contact

Further information and requests for reagents and/or resources should be directed to the lead contacts Eric Pearlman (eric.pearlman@uci.edu)

#### Materials availability

This study did not generate any new materials.

#### Data and code availability

The published article includes all datasets generated or analyzed during this study.

### EXPERIMENTAL MODEL AND SUBJECT DETAILS

#### Ethics statement

All animal studies described in this manuscript were approved by the University of California, Irvine’s IACUC committee (approved protocol: AUP-18–085). Animals were monitored twice daily for signs of distress or discomfort. Animals determined to be in distress were humanely euthanized by CO_2_ asphyxiation followed by cervical dislocation, as approved by the UCI IACUC.

#### Mice

Male and female C57BL/6J mice aged 6–8 weeks were purchased from The Jackson Laboratory (Bar Harbor, ME). All gene knock-out mice are on a C57BL/6 background. *Gsdmd*^−/−^ mice were provided by Dr. Russell Vance (University of California, Berkeley). *Nlrp3*^−/−^ mice were generated by Millennium pharmaceuticals (Cambridge, MA). *Il1a*^−/−^, *Il1b*^−/−^ and *Il1a*^−/−^*Il1b*^−/−^ mice were originally generated by Dr. Iwakura (University of Tokyo) as described (Horai et al., 1998) and were graciously provided by Dr. Obar (Dartmouth, New Hampshire; *Il1a*^−/−^, and *Il1a*^−/−^*Il1b*^−/−^), and Dr. Núñez (University of Michigan Medical School; *Il1b*^−/−^). Mice were bred under IACUC approved conditions, and all animals were housed in the University of California, Irvine vivarium. Age-matched, male and female mice were used for all experiments.

#### Fungal strains

Virulent *Aspergillus fumigatus* strain CEA10 was provided by Dr. Cramer (Dartmouth, New Hampshire), generated as previously described (Caffrey-Carr et al., 2017). Frozen glycerol stocks were maintained at −80°C and were grown on Sabouraud Dextrose agar plates (Fisher Scientific) at 37°C with 5% CO_2_.

#### Aspergillus fumigatus peritoneal inflammation model

Conidia from *A. fumigatus* strain CEA10 were incubated in SD broth until they germinated and expressed cell surface β-Glucan. Conidia were then heat-killed, and 1×10^7^ were resuspended in 500uL, then injected into the peritoneal cavity of 6- to 10-week-old age-and sex-matched mice. After 4, 6, 8, 12, or 20 hours, total peritoneal cells were collected by intraperitoneal lavage. Cells were kept on ice until processed.

#### Cell lines

The HEK-blue IL-1R1 cell-line used in this study is a commercially made cell line by InvivoGen (Cat# hkb-il1r). No sex of the cells is reported and cell authentication can be viewed under the ‘‘Data’’ PDF provided on the manufacturer’s website. Cell vials were stored in a liquid nitrogen chamber until use. For experiments, cells were transferred to a T-75 TC treated flask (Olympus) with 10 mL of warm media supplemented with 1X HEK-Blue Selection (InvivoGen). Flasks were then incubated at 37°C with 5% CO_2_. Media was replaced twice a week and cells were passaged once 70%–80% confluency was reached. Sterile PBS was used to lift cells for passaging or prepping for assays.

#### Source materials for *in vitro* studies

Bone-marrow-derived dendritic cells, peritoneal neutrophils, and peritoneal macrophages used for *in vitro* experiments in this study were all isolated from mice described above. Both female and male animals were used and were 6–10 weeks of age.

### METHOD DETAILS

#### Preparation of conidia

*Aspergillus fumigatus* was incubated on SD agar plates at 37°C for 3–5 days. To harvest conidia, 10 mL of PBS containing 0.00025% Tween-20 were added to each plate, and plastic sterile scrapers were used to collect the conidia. Conidia suspensions were then filtered and centrifuged at 500× g. Supernatant was decanted and conidia were resuspended in 5 mL sterile 1× PBS (Corning), and counted using a hemocytometer. To facilitate germination, 1×10^7^/mL conidia were incubated in 200 mL of sterile Sabouraud Dextrose broth (Fisher Scientific) at 37°C with agitation for 3–4hr until ~80% showed germination by light microscopy (seen as budding of conidia and loss of spherical shape), indicating that they express cell wall β–glucan on the surface. Germinating conidia were centrifuged at 500× g for 5 minutes. Supernatant was decanted and swollen conidia were resuspended in 1× PBS at 1×10^7^ per 500 μL. Conidia were placed in a 50 mL centrifuge tube and were heat killed by submerging tubes in boiling water for 5 minutes. Heat-killed swollen conidia were stored at 4°C until *in vivo* injections.

#### Flow cytometry

Total cell numbers were counted, and cells were stained using mouse Ly6G-BV510 (clone 1A8, BioLegend), Ly6C-PE-Cy7 (clone HK1.4, BioLegend), and F4/80-FitC (clone BM8, BioLegend), IL-1α-PE (clone ALF-161, BioLegend), IL-1β-APC antibodies (clone NJTEN3, Thermo Fisher), and amine-reactive fixable viability dye e780 (Invitrogen, Thermofisher). Cell surface staining was performed at 4°C for 20 minutes. Cells were fixed for 15 minutes at 4°C and then permeabilized for intracellular stain (30 minutes) with Cytofix/Cytoperm kit (BD Biosciences). Flow cytometry and analysis was conducted on ACEA Novocyte instrument and Novoexpress software, respectively. The frequency of Ly6G+ neutrophils, F4/80+ macrophages, and F4/80- Ly6G- Ly6C^hi^ monocytes were multiplied by total cell count to get cell numbers of each cell type.

#### Bone-marrow-derived dendritic cells

Hind leg femurs and tibias were dissected from mice and were cleaned of tissue. The bones were clipped to expose the bone marrow. Up to four bones were placed in a 0.6 mL microcentrifuge tube (Genesee Scientific) which had a hole pierced through the bottom of it by an 18-gauge needle (Fisher Scientific). This 0.6 mL microcentrifuge tube with bones was capped and placed in a 1.5 mL microcentrifuge tube (Genesee Scientific) and was centrifuged at 10,000× g for approximately 10 s (just enough time for centrifuge to get up to speed, and immediately stopped). Bone marrow was then present in 1.5 mL microcentrifuge tube while the emptied bones remained in the 0.6 mL microcentrifuge tube. The 0.6 mL microcentrifuge tube was discarded, and bone marrow cells were resuspended in 1 mL of warmed RPMI (GIBCO). Cells were then placed in T-75 TC culture flasks with 10 mL of RPMI (GIBCO) supplemented with 10% FBS (Corning), 1% penicillin-streptomycin (GIBCO), 1% non-essential amino acids (GIBCO), 1% sodium pyruvate (GIBCO), and 10 ng/mL granulocyte-macrophage colony-stimulating factor (StemCell Technologies). Cells were incubated at 37°C with 5% CO_2_ for 7 days. Media was replaced every other day. On day 7, semi-adherent cells were gently washed with PBS to lift, and cells were stained for CD11c, Ly6G, F4/80, and viability dye. CD11c+ Ly6G- F4/80- dendritic cells were isolated by BD FACS Aria Fusion flow cytometer.

#### Peritoneal neutrophils and macrophages

##### Neutrophils

Intraperitoneal injection of 1 mL 9% casein (Sigma Aldrich) was used to induce sterile inflammation in 6- to 10-week-old mice 16 hours prior to collection, and boosted again 3 hours prior to lavage. To collect cells, the peritoneal cavity was flushed with 10 mL PBS and the lavage fluid was centrifuged at 300× g for 5 minutes. Neutrophils were isolated using a negative bead selection kit (StemCell Technologies), which routinely yields > 95% neutrophils. Cells were diluted to 2.5 × 10^6^ neutrophils/mL and were plated in RPMI (GIBCO) supplemented with 10% FBS (Corning), 1% penicillin-streptomycin (GIBCO), 1% non-essential amino acids (GIBCO), 1% sodium pyruvate (GIBCO), and 10 ng/mL granulocyte-macrophage colony-stimulating factor (StemCell Technologies).

##### Macrophages

Intraperitoneal injection of 1 mL 9% casein (Sigma Aldrich) was used to induce sterile inflammation in 6- to 10-week-old mice 4 days prior to collection of peritoneal cells. The peritoneal cavity was flushed with 10 mL PBS and the lavage fluid was centrifuged at 300× g for 5 minutes to collect peritoneal cells. Cells were plated overnight at 1×10^6^ cells/mL in DMEM (GIBCO) with 10% FBS (Corning), 1% penicillin-streptomycin (GIBCO, Life Technologies), 1% non-essential amino acids (GIBCO), 1% sodium pyruvate (GIBCO), and 10 ng/mL granulocyte-macrophage colony-stimulating factor (StemCell Technologies) for adherence. Non-adherent cells were aspirated the next day, and each well was washed with PBS before adding fresh media. Adherent macrophages were lifted using Cell Stripper (Corning, NY).

##### *In vitro* stimulation

BMDCs and macrophages were incubated at 5 × 10^5^/mL (neutrophils at 2.5 × 10^6^/mL) with either 100 μg/mL curdlan (Sigma Aldrich), 500 ng/mL ultrapure *E. coli* LPS (Invivogen), or LPS + 3 mM extracellular ATP (Sigma Aldrich) added in the last hour of incubation. All cells were incubated at 37°C with 5% CO_2_.

#### Flow cytometry and ImageStream™ flow cytometry

Cells were stained using the following fluorophore conjugated anti-mouse antibodies: WGA-488 (Invitrogen), Ly6G-FitC (clone 1A8, BioLegend), Ly6G-BV510 (clone 1A8, BioLegend), Ly6G-PE (clone 1A8, BioLegend), CD11c-BV605 (clone N418, BioLegend), F4/80-FitC (clone BM8, BioLegend), F4/80-PE (clone BM8, BioLegend), CD11b-PE (clone M1/70, BioLegend), Ly6C-PE Cy7 (clone HK1.4, BioLegend), CD63-APC (clone NVG-2, BioLegend), CD9-APC (clone MZ3, BioLegend), IL-1α-PE (clone ALF-161, BioLegend), and IL-1β-APC (clone NJTEN3, ThermoFisher Scientific). Antibodies were diluted in wash buffer (PBS with 1% BSA and 2 mM EDTA). Cells were stained for 20 minutes at 4°C, washed with wash buffer, fixed for 15 minutes in BD Biosciences cytofix/cytoperm, and permeabilization prior to intracellular staining. ACEA Novocyte was used for flow cytometry, and Novoexpress software was used for subsequent analysis. AMNIS ImageStream was used for imaging flow cytometry and the AMNIS IDEAS software was used to calculate the colocalization coefficient.

#### Western blot

BMDCs, peritoneal macrophages, or peritoneal neutrophils were lysed with 1X CST lysis buffer (Cell Signaling). For neutrophils, DFP (Sigma Aldrich) was added to the lysis buffer to inhibit any protease activity. BCA assay kit (Thermo Fisher) was used to determine protein concentration from lysates. Twenty μg of protein from lysates mixed with 1X SDS (from 5X stock), and Ultrapure water (Invitrogen) were boiled for 10 minutes at 95°C on a heating block. Samples were loaded into 4%–20% mini-PROTEAN, 10-well, 50 μl TGX precast SDS-PAGE gels (Bio-rad). Gels were run in 1X TAE buffer at a constant 110V. Proteins were transferred onto a nitrocellulose membrane using Bio-rad Trans-blot Turbo transfer system. The membrane was blocked with 5% milk for 1 hour at RT, and incubated with rabbit anti-mouse GSDMD (EPR20859, Abcam) or mouse anti-β-actin (Santa Cruz Biotechnology) diluted in 5% milk and incubated at 4°C overnight on a rocker. Membranes were washed with 1X TBST buffer 3× for 10 minutes. HRP-conjugated secondary antibodies against rabbit or mouse IgG (Cell Signaling) were diluted in 5% milk and incubated at RT for 1 hour. West Femto Maximum Supersignal (Thermo Fisher) was used to enhance signal before the membrane was imaged by the Chemidoc (BioRad) instrument.

#### Immunofluorescence and confocal microscopy

Stimulated neutrophils were collected after 6 hours and stained with Ly6G-FITC antibody (clone 1A8, BioLegend) for 20 minutes. Cells were washed and fixed with 4% PFA (BD Biosciences) overnight. To permeabilize fixed cells, 0.1% TritonX (Fisher Scientific) was used. Cells were incubated with 10% normal donkey serum (NDS, Jackson ImmunoResearch) for 1 hour before addition of primary antibodies: ALF-161 Armenian hamster anti-mouse IL-1α (Fisher Scientific), and rabbit anti-mouse CD63 (clone EPR21151, Ab-cam). Primary antibodies were incubated overnight at 4°C. Cells were washed twice with FACS buffer (PBS with 1% BSA and 2 mM EDTA). AF546 goat anti-hamster IgG (ThermoFisher Scientific) and AF647 donkey anti-rabbit IgG (ThermoFisher Scientific) secondary antibodies were added and incubated at room temperature for 30 minutes. 4 μL of cells were mixed with 4 μL of Vectashield® antifade mounting media with DAPI (Vector Laboratories) and plated on a coverslip. Imaging was done using an LSM700 confocal microscopy (Optical Biology Core, UCI, Leica LSM700) and analyzed with Zen software.

#### Cytokine analysis

***ELISA*** was used for quantification of cytokines in cell-free supernatant and lavage fluid. Cells were centrifuged at 300× g for 5 minutes and the supernatant was collected and stored at −80°C. Murine IL-1α and IL-1β ELISA kits were purchased from R&D Systems. A Biotek Cytation-5 plate reader was used to quantify concentrations.

***HEK-blue IL-1R1 cell-line*** used to measure IL-1α and IL-1β bioactivity was purchased from InvivoGen. For each experiment, 2.8×10^5^ HEK IL-1R1 reporter cells/mL in DMEM (GIBCO) complete media were seeded in 180 μL per well. Twenty μL of each sample was added in duplicates without neutralizing antibodies, with anti-IL-1α neutralizing antibodies (Fisher Scientific), anti-IL-1β neutralizing antibodies (R&D Systems), or both and incubated overnight at 37°C with 5% CO_2_. The supernatant was collected and incubated with QUANTI-Blue (InvivoGen) for 30 minutes. SEAP detection and concentration calculations were measured on the Biotek Cytation-5 instrument. IL-1α and IL-1β concentrations were calculated based on a set of standards of known bioactive IL-1α and IL-1β concentrations and presented as pg/mL.

#### Lactate dehydrogenase (LDH) assay for cell death

Promega CytoTox 96® Non-radioactive Cytotoxicity Assay was used to measure LDH release in cell-free supernatant. Released LDH in culture supernatants was measured with a 30-minute coupled enzymatic assay, which results in conversion of a tetrazolium salt (INT) into a red formazan product. Maximum LDH control was prepared by lysing the same concentration of cells in each experiment with lysis buffer from the Promega kit for 15 minutes prior to collection. LDH read was measured at 490nm with a Biotek Cytation-5 instrument. The % of max LDH release was calculated by dividing the LDH read from each sample by the maximum LDH control read. The maximum LDH control was prepared for each individual experiment.

#### Propidium iodide (PI) uptake assay for plasma membrane permeability

Cells were plated in a black-sided, optically clear flat bottom 96-well plate (Corning). PI (Alfa Aesar) was added to each well at 1:10,000 dilution in PBS. Cells were incubated in the Biotek Cytation-5 instrument at 37°C with 5% CO_2_. Red fluorescence reads at 590/640nm were measured every 1 minute for 5 minutes for background measurement before adding stimulation. Duplicate wells of cells were primed with 500 ng/mL *E. coli* LPS (Invivogen) or 100 μg/mL curdlan (Sigma Aldrich) for 6 hours. Three mM extracellular ATP (Sigma Aldrich) was added to LPS primed cells in the last hour of incubation in the LPS+ATP conditions. Fluorescence measurements were taken at 1-minute intervals throughout stimulation.

#### Extracellular vesicle isolation

Peritoneal neutrophils were cultured in RPMI (GIBCO) supplemented with 10% exosome-depleted FBS (Systems Biosciences, SBI) containing 1% penicillin-streptomycin, 1% non-essential amino acids, 1% sodium pyruvate, and 10 ng/mL granulocyte-macrophage colony-stimulating factor (StemCell Technologies). After 6 hours of stimulation, 200 μL of ExoQuick-TC reagent (System Biosciences, SBI) was added to 1 mL of cell-free supernatant from cultured neutrophils and was inverted to mix. The supernatant was incubated overnight at 4°C, and extracellular vesicles were recovered after centrifugation (1500× g for 30 minutes at 4°C). Supernatant was aspirated and dry pellets were resuspended in 1 mL PBS and frozen at −20°C for short-term storage.

#### Nanoparticle tracking analysis and flow cytometry of EVs

A Malvern Nanosight NS300 was used for nanoparticle tracking analysis of isolated EVs. EVs suspended in PBS (700uL) was used to measure 3 technical replicates, measuring size distribution and concentration from 60 s video (approximately 10^7^-10^8^ particles/mL concentration) with constant syringe flow. For flow cytometry analysis, EVs were stained and detection threshold on the ACEA Novocyte instrument was lowered to 1000 on the FSC. For ELISA, resuspended EV pellets were lysed with 1% Triton X (Fisher Scientific) and loaded into a 96-well plate followed by the standard ELISA protocol from R&D Systems.

#### Exosome isolation by differential ultracentrifugation

Neutrophils stimulated for 6 hours with LPS, LPS/ATP, or curdlan were pelleted at 300× g, 4°C for 5 minutes. The cell-free supernatant was then centrifuged at 2000× g, 4°C for 10 minutes to pellet dead cells and debris. Next, the supernatant was centrifuged at 10,000× g and the pellet (microvesicles and cell debris) was discarded. The remaining supernatant was ultracentrifuged at 100,000× g, 4°C for 70 minutes and the pellet was washed three times with PBS before resuspension in PBS for further analysis.

### QUANTIFICATION AND STATISTICAL ANALYSIS

Statistical analysis was determined by ordinary one-way ANOVA with Dunnett’s multiple comparisons test, or by 2-way ANOVA with Tukey’s multiple comparisons test (detailed in figure legends) using GraphPad Prism software. Error bars indicate mean ± SEM and p values less than 0.05 were considered significant.

## Supplementary Material

1

2

## Figures and Tables

**KEY RESOURCES TABLE:** 

REAGENT or RESOURCE	SOURCE	IDENTIFIER

Antibodies		

Brilliant Violet 510 anti-mouse Ly-6G Antibody, clone 1A8	BioLegend	RRID:AB_2562937; Cat#127633
PE/Cyanine7 anti-mouse Ly-6C Antibody, clone HK1.4	BioLegend	RRID:AB_1732082; Cat#128018
FITC anti-mouse F4/80 Antibody, clone BM8	BioLegend	RRID:AB_893502; Cat#123108
PE anti-mouse IL-1α Antibody, clone ALF-161	BioLegend	Cat#503203
IL-1 beta (Pro-form) Monoclonal Antibody (NJTEN3), APC	ThermoFisher Scientific	RRID:AB_10670739; Cat#17-7114-80
Fixable Viability Dye eFluor 780	eBioscience	Cat#65-0865-18
Wheat Germ Agglutinin, Alexa Fluor 488 Conjugate	Invitrogen	Cat#W11261
FITC anti-mouse Ly-6G Antibody, clone 1A8	BioLegend	RRID:AB_1236494; Cat#127606
PE anti-mouse Ly-6G Antibody, clone 1A8	BioLegend	RRID:AB_1186099; Cat#127608
Brilliant Violet 605 anti-mouse CD11c Antibody, clone N418	BioLegend	RRID:AB_2562415; Cat#117334
PE anti-mouse F4/80 Antibody, clone BM8	BioLegend	RRID:AB_893486; Cat#123110
PE anti-mouse/human CD11b Antibody, clone M1/70	BioLegend	RRID:AB_312791; Cat#101208
APC anti-mouse CD63 Antibody, clone NVG-2	BioLegend	RRID:AB_2565496; Cat#143906
APC anti-mouse CD9 Antibody, clone MZ3	BioLegend	RRID:AB_2783070; Cat#124812
Recombinant Anti-GSDMD antibody [EPR20859]	Abcam	RRID:AB_2888940; Cat#ab219800
Actin Antibody (C-2)	Santa Cruz Biotechnology	RRID:AB_626630; Cat#sc-8432
Mouse IL-1 beta/IL-1F2 Antibody	R&D Systems	RRID:AB_354347; Cat#AF-401-NA
Anti-mouse IgG, HRP-linked Antibody	Cell Signaling Technology	RRID:AB_330924; Cat#7076S
Anti-rabbit IgG, HRP-linked Antibody	Cell Signaling Technology	RRID:AB_2099233; Cat#7074S
IL-1α Hamster anti-Mouse, PE, Clone: ALF-161, BD	Fisher Scientific	Cat#BDB559810
Recombinant Anti-CD63 antibody [EPR21151]	Abcam	Cat#ab217345
Goat anti-Rabbit IgG (H+L) Highly Cross-Adsorbed Secondary Antibody, Alexa Fluor 546	ThermoFisher Scientific	RRID:AB_2534093; Cat#A-11035
Donkey anti-Rabbit IgG (H+L) Highly Cross-Adsorbed Secondary Antibody, Alexa Fluor 647	ThermoFisher Scientific	RRID:AB_2536183; Cat#A-31573

Bacterial and virus strains		

*Aspergillus fumigatus,* Strain: CEA10	Dr. Robert Cramer, Dartmouth, New Hampshire	N/A

Chemicals, peptides, and recombinant proteins		

Casein sodium salt from bovine milk	Sigma Aldrich	CAS#9005-46-3; Cat#C8654-500G
Mouse Recombinant GM-CSF (*E. coli*-expressed)	StemCell Technologies, Inc.	Cat#78017.1
Curdlan from *Alcaligenes faecalis*	Sigma Aldrich	CAS#54724-00-4; Cat#C7821-5G
LPS-EK ULTRAPURE (Ultrapure lipopolysaccharide from *E. coli* K12)	InvivoGen	Cat#tlrl-peklps
Adenosine 5′-triphosphate disodium salt hydratemicrobial, BioReagent, suitable for cell culture, ≥ 99% (HPLC)	Sigma Aldrich	CAS#34369-07-8; Cat#A6419-1G
Normal Donkey Serum	Jackson Immunoresearch	RRID:AB_2337258; Cat#017-000-121
Regular Fetal Bovine Serum, Heat Inactivated	Corning	Cat#MT35011CV
VECTASHIELD® HardSet Antifade Mounting Medium with DAPI	Vector Laboratories	Cat#H-1500-10
QUANTI-Blue™ Solution (Alkaline phosphatase detection medium - Liquid form)	InvivoGen	Cat#rep-qbs2
Propidium iodide, 1mg/ml aqueous soln.	Alfa Aesar	CAS#25535-16-4; Cat#J66584-AB
Exosome-depleted FBS Media Supplement	System Biosciences (SBI)	Cat#EXO-FBS-250A-1
ExoQuick-TC	System Biosciences (SBI)	Cat#EXOTC10A-1
Diisopropylfluorophosphate (DFP)	Sigma Aldrich	CAS#55-91-4; Cat#D0879-1G
Cell Lysis Buffer (10X)	Cell Signaling Technology	Cat#9803S
HEK-Blue™ Selection (Antibiotics for maintenance of HEK-Blue Cells)	InvivoGen	Cat#hb-sel

Critical commercial assays		

EasySep™ Mouse Neutrophil Enrichment Kit	StemCell Technologies, Inc.	Cat#19762
Pierce BCA Protein Assay Kit - Reducing Agent Compatible	ThermoFisher Scientific	Cat#23250
SuperSignal West Femto Maximum Sensitivity Substrate	ThermoFisher Scientific	Cat#34095
Mouse IL-1 alpha/IL-1F1 Duoset ELISA	R&D Systems	Cat#DY400-05
Mouse IL-1 beta/IL-1F2 Duoset ELISA	R&D Systems	Cat#DY401 -05
CytoTox 96® Non-Radioactive Cytotoxicity Assay	Promega	Cat#G1780
BD Cytofix/Cytoperm Fixation/Permeablization Kit	BD Biosciences	RRID:AB_2869008; Cat#554714

Experimental models: Cell lines		

HEK-Blue™ IL-1R Cells (HEK293 reporter cells for human and murine IL-1α & IL-1β cytokines)	InvivoGen	Cat#hkb-il1r

Experimental models: Organisms/strains		

Mouse: C57BL/6J	The Jackson Laboratory (JAX)	Cat#000664
Mouse: GSDMD^−/−^, C57BL/6J background	Dr. Russel Vance, University of California, Berkeley	N/A
Mouse: NLRP3^−/−^, C57BL/6J background	Millennium Pharmaceuticals, Cambridge, MA	N/A
Mouse: IL-1α^−/−^, C57BL/6J background	Dr. Y. Iwakura, University of Tokyo	N/A
Mouse: IL-1β^−/−^, C57BL/6J background	Dr. Y. Iwakura, University of Tokyo	N/A
Mouse: IL-1α^−/−^ IL-1β^−/−^, C57BL/6J background	Dr. Y. Iwakura, University of Tokyo	N/A

Software and algorithms		

Flowjo	Flowjo	RRID: SCR_008520
GraphPad Prism	GraphPad	RRID:SCR_002798
IDEAS	AMNIS, Luminex	N/A
NovoExpress Software	Agilent	N/A

## Highlights

- Neutrophils are an important source of IL-1α
- IL-1α secretion by dendritic cells and macrophages is dependent on NLRP3 and GSDMD
- Neutrophil secretion of IL-1α is independent of NLRP3, GSDMD, and cell death
- IL-1α secretion by neutrophils is mediated by extracellular vesicles
