# Supplementary material for: β-Glucan-stimulated neutrophil secretion of IL-1α is independent of GSDMD and mediated through extracellular vesicles
Source: Cell Rep. Author manuscript; Available in PMC 2021 Jun 8. (PMC8186457; doi:10.1016/j.celrep.2021.109139)
Supplement: 1 [file NIHMS1709019-supplement-1.pdf]

Cell Reports, Volume *35*

Supplemental information

β-Glucan-stimulated neutrophil secretion of IL-1α is independent of GSDMD and mediated through extracellular vesicles

Bridget Ratitong, Michaela Marshall, and Eric Pearlman

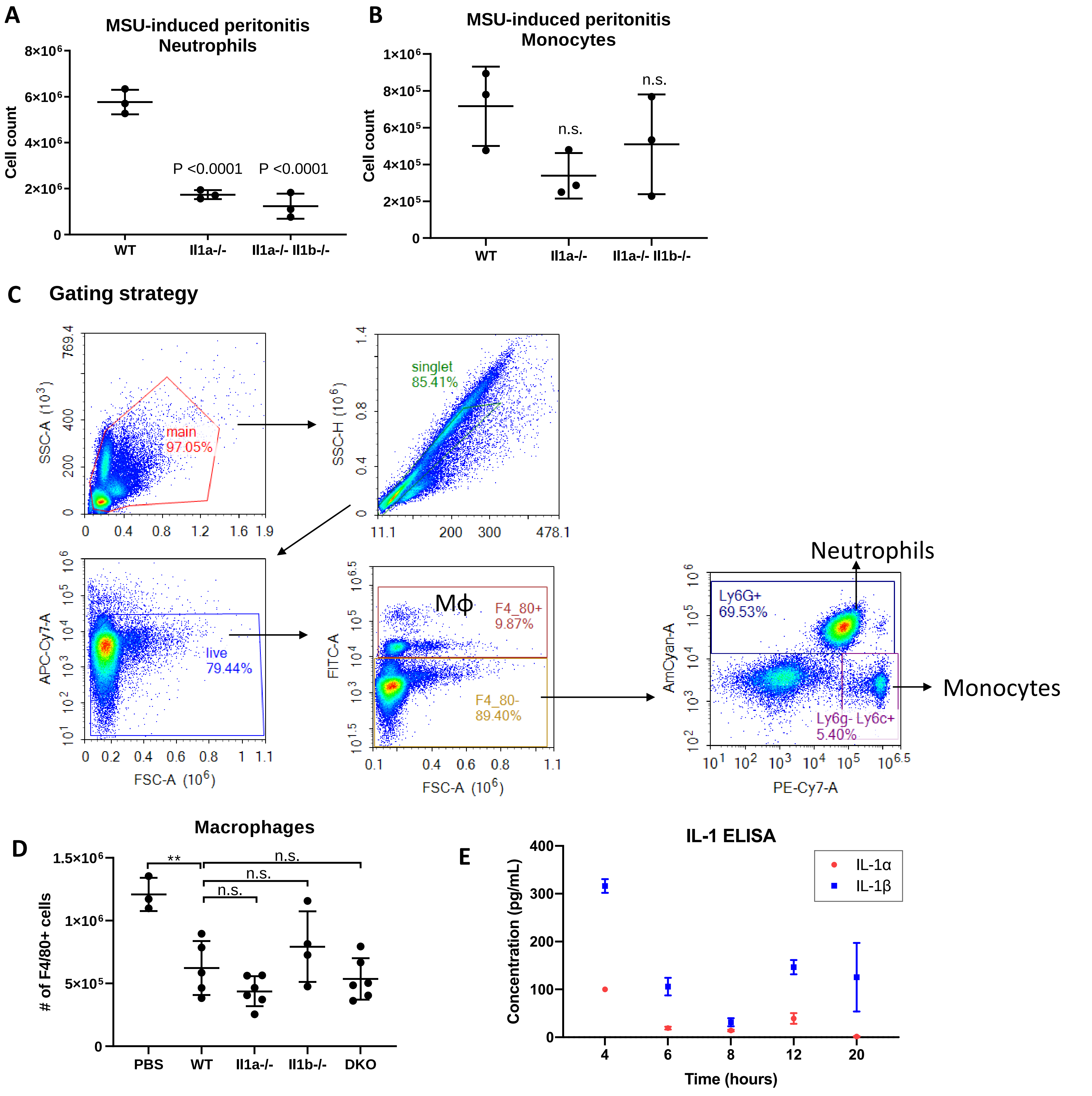

Supplementary Figure 1. IL-1α is important for neutrophil recruitment. Related to Figure 1.
(A, B) Neutrophil and monocyte cell count quantified 4h after intraperitoneal injection of 2 mg/ mouse monosodium urate (MSU) into C57BL/6 (WT), IL-1α$^{-/-}$, and IL-1α$^{-/-}$/IL-1β$^{-/-}$ mice (n=3).
(C) Representative flow cytometry plots demonstrating gating strategy for live, singlet F4/80+ macrophages, F4/80- Ly6G+ neutrophils, and F4/80- Ly6G- Ly6C+ monocytes.
(D) F4/80+ macrophage cell counts from the peritoneal cavity of mice injected with swollen, heat killed *A. fumigatus* conidia.
(E) IL-1α and IL-1β concentration from the peritoneal lavage fluid at multiple time points following injection of heat-killed *A. fumigatus* conidia (n=3).

Supplementary Figure 2

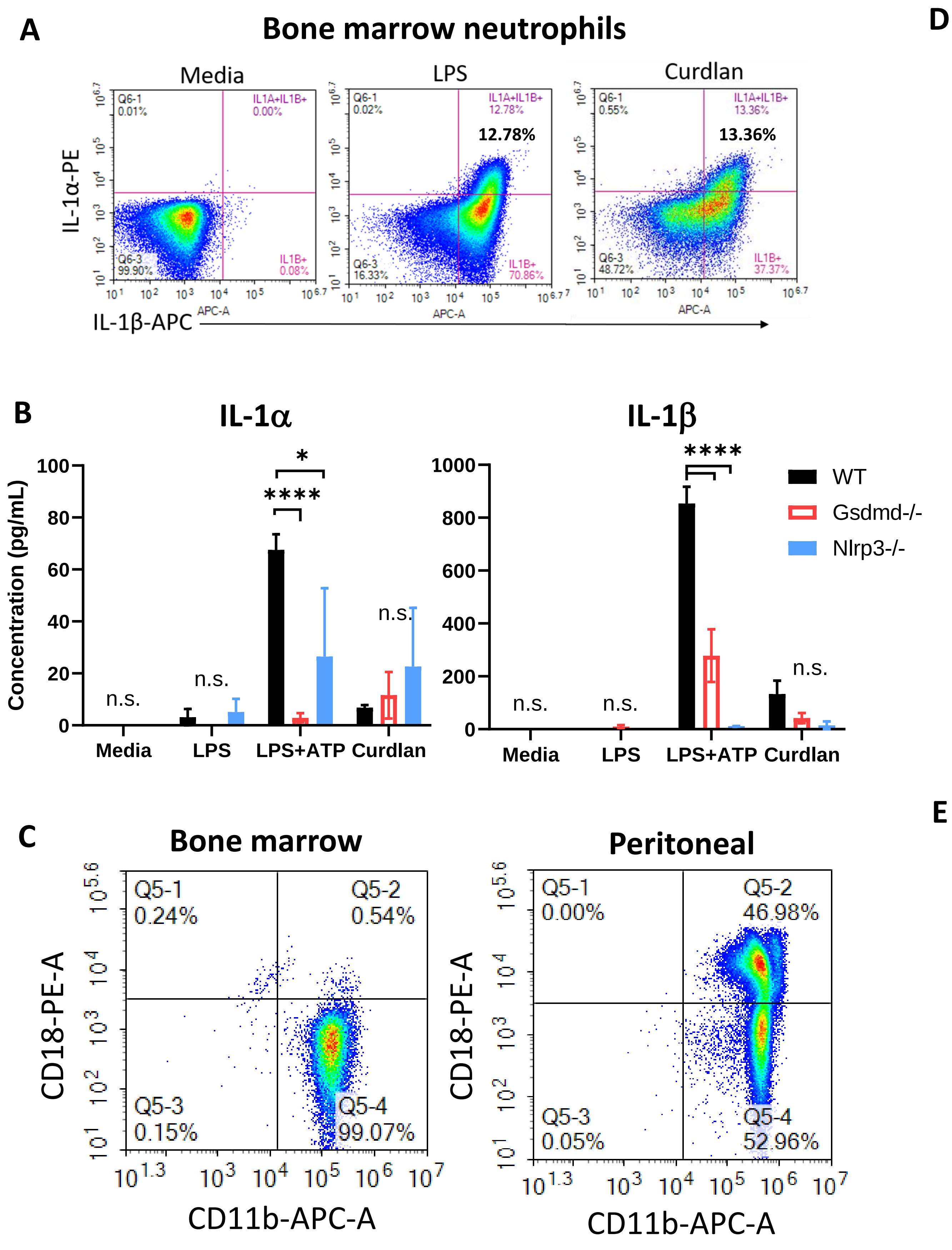

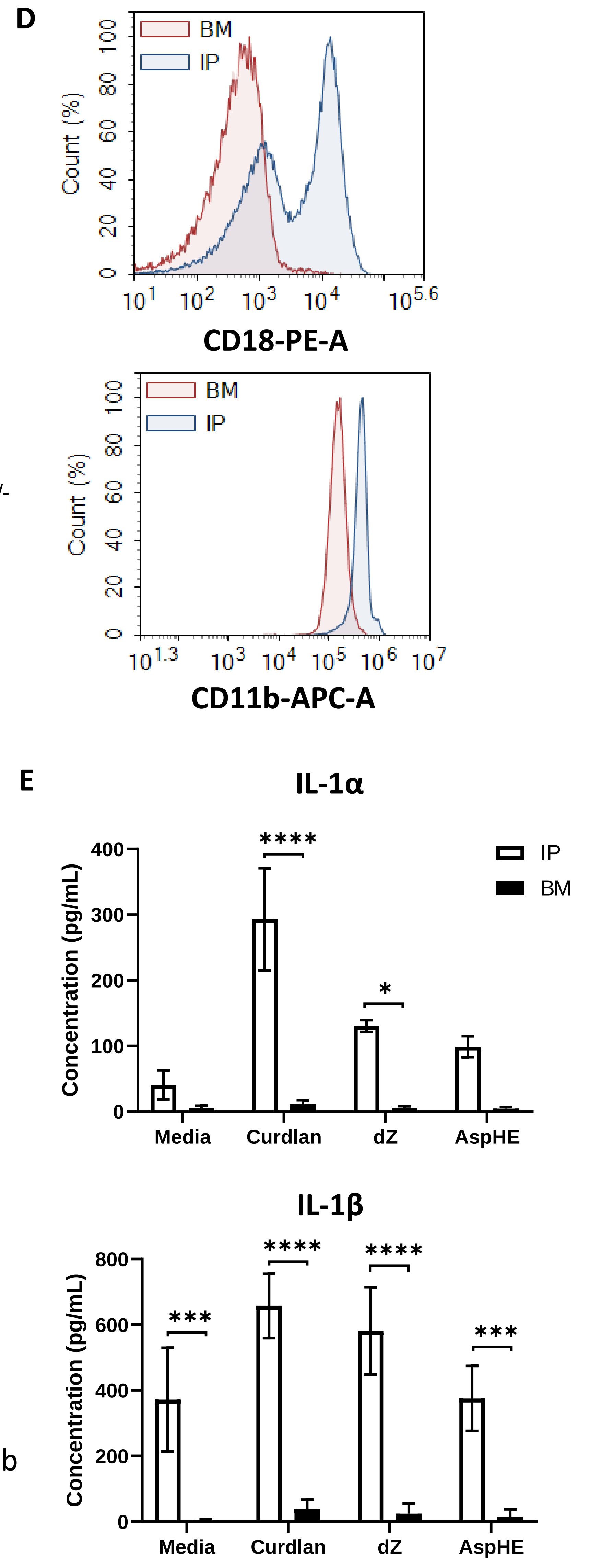

Supplementary Figure 2. IL-1α and IL-1β production by bone marrow (BM) neutrophils. Related to Figure 2.

(**A, B**) Intracellular and secreted IL-1α and IL-1β following 6h stimulation with LPS, LPS/ATP (ATP added in the last hour only) or curdlan.

(**C, D**) Representative scatter plots and histogram of CD18 and CD11b on the plasma membrane of neutrophils from the peritoneal cavity following casein injection, or bone marrow of naïve mice.

(**E**) β-glucan secretion of IL-1α and IL-1β by BM compared with peritoneal (IP) neutrophils in response to curdlan, depleted zymosan (dZ), or *A. fumigatus* hyphal extract (AspHE).

Supplementary Figure 3

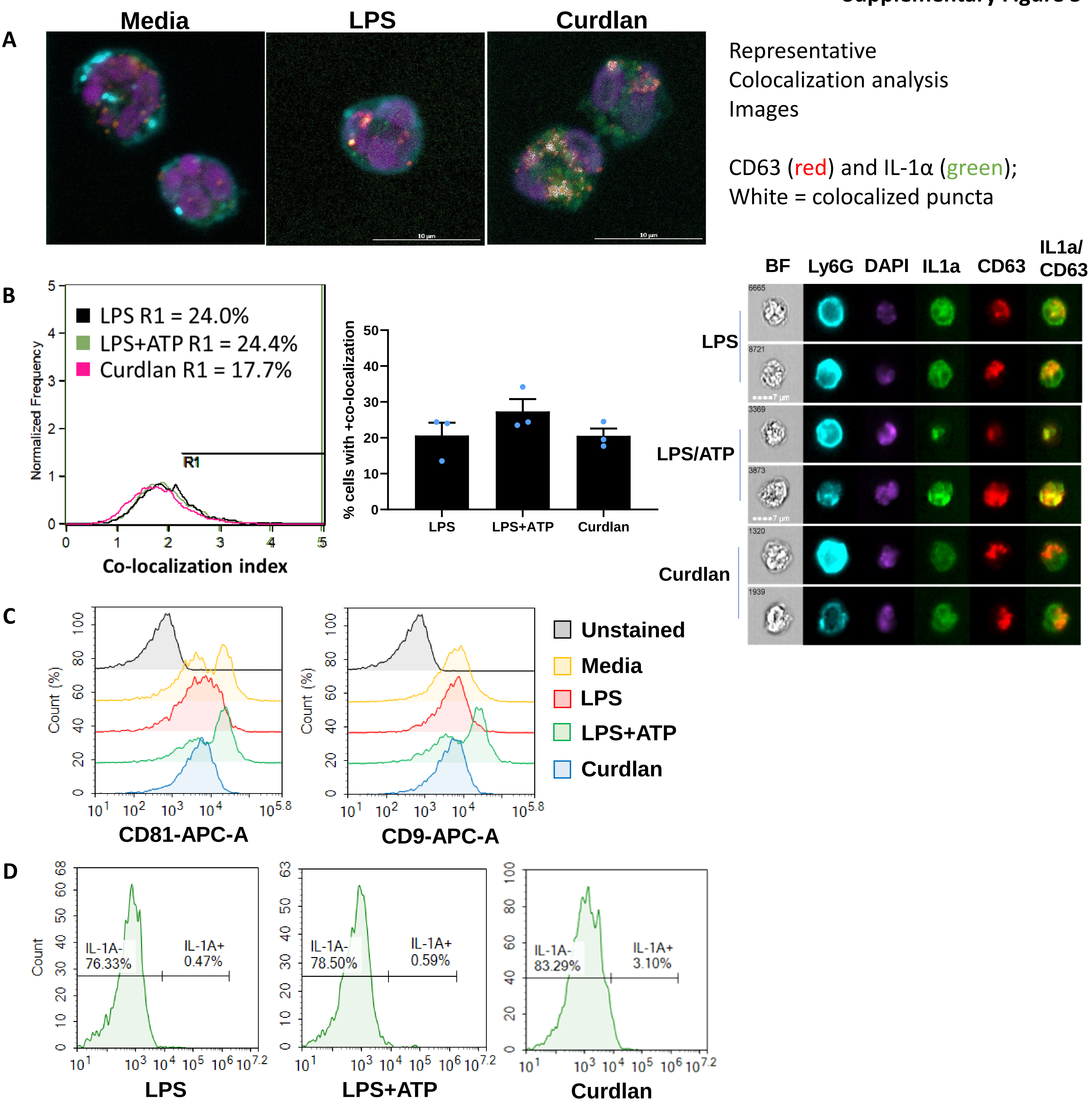

Supplementary Figure 3. Characterization of extracellular vesicles from peritoneal neutrophils. Related to Figure 4.

(**A**) Co-localization analysis by ImageJ of CD63 and IL-1α in unstimulated, LPS- or curdlan-stimulated peritoneal neutrophils. CD63 (red) and IL-1α (green); colocalization of CD63 and IL-1α is shown in white.

(**B**) Representative Amnis ImageStream™ images (left) showing plasma membrane Ly6G and intracellular CD63/IL-1a. Colocalization coefficient of CD63/IL-1a (center panel), and percent co-localization (right panel) of neutrophils stimulated with LPS, LPS/ATP, or curdlan (n=3)

(**C**) Flow cytometry of representative isolated EVs stained with exosome markers CD81 or CD9

(**D**) Representative flow histogram showing no IL-1α on membrane of isolated EVs

Supplementary Figure 4

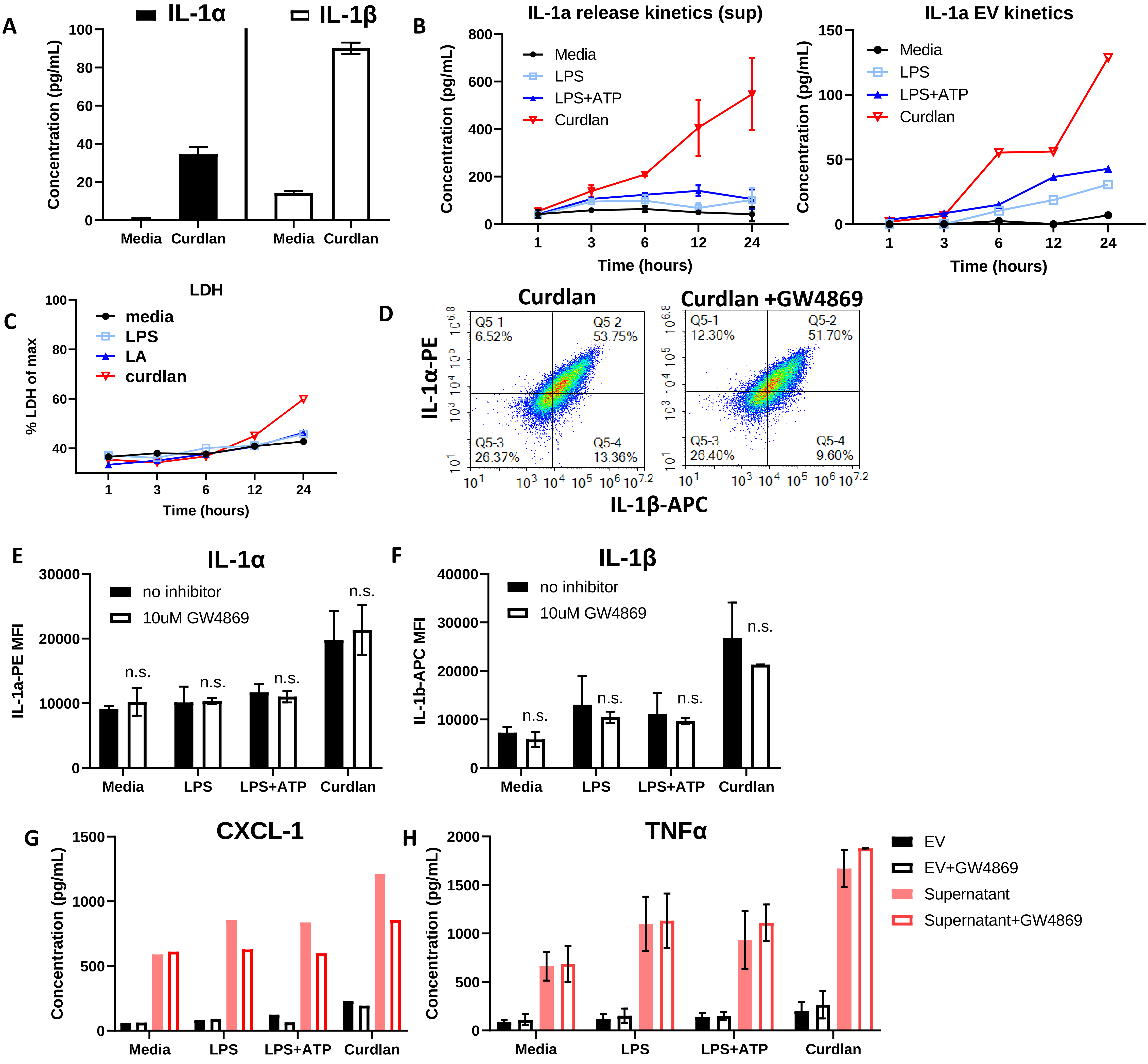

Supplementary Figure 4. IL-1 secretion through EVs increase over time and GW4869 does not affect IL-1 production. Related to Figure 4.

(**A**) IL-1α and IL-1β produced by neutrophil exosomes isolated by differential ultracentrifugation (pellet at 100,000xg) and lysed by Triton-X100

(**B**) Total IL-1α (left) and lysed EV IL-1α (right) time course of secretion by neutrophils stimulated with LPS, LPS/ATP, or curdlan for 1, 3, 6, 12, and 24 hours.

(**C**) LDH release time course from neutrophils stimulated for 1, 3, 6, 12, and 24 hours.

(**D)** Representative flow cytometry plots of intracellular IL-1α-PE and IL-1β-APC in GW4869-pre treated neutrophils showing no effect of the inhibitor on intracellular staining.

(**E,F**) MFI quantification of intracellular IL-1α-PE and IL-1β-APC in GW4869-treated neutrophils

(**G, H**) CXCL1 and TNFa is not present in EVs or blocked by the exosome inhibitor GW4869
